# Determination of Free Histidine in Complex Hair Care Products with Minimum Sample Preparation Using Cation-Exchange Chromatography and Post Column Derivatization

**DOI:** 10.3390/molecules28020888

**Published:** 2023-01-16

**Authors:** Apostolia Tsiasioti, Constantinos K. Zacharis, Paraskevas D. Tzanavaras

**Affiliations:** 1 Laboratory of Analytical Chemistry, School of Chemistry, Faculty of Sciences, Aristotle University of Thessaloniki, GR-54124 Thessaloniki, Greece; 2 Laboratory of Pharmaceutical Analysis, Department of Pharmaceutical Technology, School of Pharmacy, Aristotle University of Thessaloniki, GR-54124 Thessaloniki, Greece

**Keywords:** histidine, high-pressure liquid chromatography, postcolumn derivatization, fluorescence, hair care products

## Abstract

In this communication, we describe the first analytical method for the determination of free histidine in hair care products (shampoos and conditioners). Cation-exchange chromatography combined with postcolumn derivatization and fluorimetric detection enabled the accurate (recovery: 83.5–114.8%) and precise (2.4–5.6% RSD) determination of free histidine without matrix interferences at concentration levels down to 1.5 mg kg^−1^. Real commercially available samples were found to contain the amino acid at levels ranging between 70 and 535 mg kg^−1^.

## 1. Introduction

Recent published research has proven experimentally that the presence of copper traces in human hair promotes damage (color change, split ends, loss of shine, etc.) [1,2] catalyzed by UV exposure [3]. Copper ions mainly from exogenous tap water sources were determined in the sulfur-poor endo-cuticle [4], while possible action mechanisms were identified ranging from the formation of reactive oxygen species to participation in the oxidation of lipids [5]. A potential solution to the latter issues is the addition of copper chelators to daily hair care products (shampoos and conditioners) that proved to improve hair health by reducing the effect of UV exposure [6].

Well-known metal chelators such as N,N’-ethylenediamine disuccinic acid were found to be effective for copper removal in shampoos [3] but they were not applicable for conditioners, causing instability of the products due to their negatively charged functional groups. Although not an obvious alternative, the amino acid histidine [6] offers (i) increased chelating activity due to the imidazole group compared with other amino acids [7], (ii) effective chelation of copper in the presence of a significant excess of calcium and (iii) effective chelation at the typical pH of shampoos and conditioners [8].

From an analytical chemistry point of view, hair care products are considered to be complicated matrices containing numerous organic and inorganic compounds, thus requiring extra sample cleanup steps prior to the end-point analysis. Representative recent analytical applications in hair care products are presented in Table 1 [9,10,11,12,13,14,15,16,17,18]. As can be seen in the examples of Table 1, solid- or liquid-phase extraction steps are quite common even when sophisticated LC-MS instrumentation is employed, while dry ashing/wet digestion are necessary for the determination of inorganic analytes.

In the present study, we report the straightforward determination of free histidine in hair care products (shampoos and conditioners) using cation-exchange chromatography coupled to specific postcolumn derivatization and fluorimetric detection. To the best of our knowledge, this is the first analytical report for histidine in this type of matrix. The method is based on the cation-exchange separation of the analyte from the complicated substrates followed by an online postcolumn reaction with o-phthalaldehyde in the absence of nucleophilic compounds [19,20]. Validation experiments confirmed the absence of matrix effects and allowed the adaptation of very simple and convenient sample processing (dissolution–filtration–dilution).

## 2. Results and Discussion

### 2.1. Investigation of the Separation Conditions

**Figure 1:**
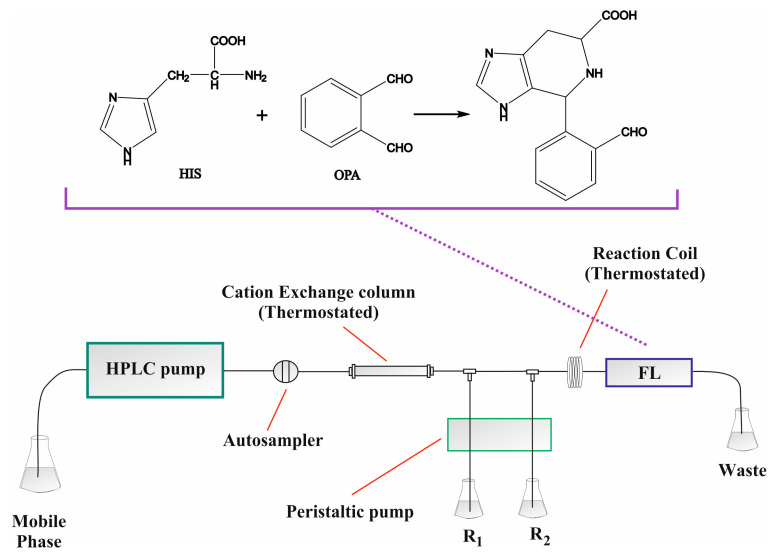
Graphical depiction of the HPLC-PCD setup for the determination of free histidine in hair care products; 5 mmol L^−1^ HNO_3_ mobile phase at 1 mL min^−1^ (60 °C), 10 mmol L^−1^ OPA, 50 mmol L^−1^ KH_2_PO_4_ (pH = 9), 0.25 mL min^−1^ flow rate for each stream, 200 cm reaction coil (60 °C), λ_ex_/λ_em_ = 340/460 nm.

Despite the negative charge of the carboxylic acid moiety, histidine can be retained on cation-exchange columns through the positively charged nitrogen atoms in its molecule. Although previous studies of our group have shown rather fast elution of the analyte, the combination with selective postseparation detection has proven to be adequate for the analysis of complex matrices [20]. It was therefore necessary to examine the chromatographic profiles of the samples prior to selection of the final separation conditions. Such preliminary investigations are necessary in order to avoid “surprises” during real sample analyses. Three histidine-containing shampoos and three histidine-containing conditioners were randomly selected and were analyzed independently using 5 mmol L^−1^ HNO_3_ as the mobile phase. Only a single peak corresponding to histidine was recorded in all cases, despite the complex background of the samples. Similar results were obtained by our group for biological material as well and can be attributed to the specific postcolumn derivatization reaction [20]. Increase in the acidity of the mobile phase led to elution of the analyte at the void volume of the HPLC and also stressed the analytical column in terms of pH tolerance. On the other hand, lower acidity (3 mmol L^−1^ HNO_3_) increased the separation cycle without any other obvious gain. No obvious gain was also recorded by using 5–10% acetonitrile in the mobile phase. The PCD conditions (concentration of the reagent, pH, buffer, temperature, etc.) were adopted from previous experimental work from our group without changes as they provided adequate selectivity [20]. A graphical depiction of the HPLC-PCD setup (including the derivatization reaction) can be seen in Figure 1.

### 2.2. Investigation of the Extraction Conditions

**Figure 2:**
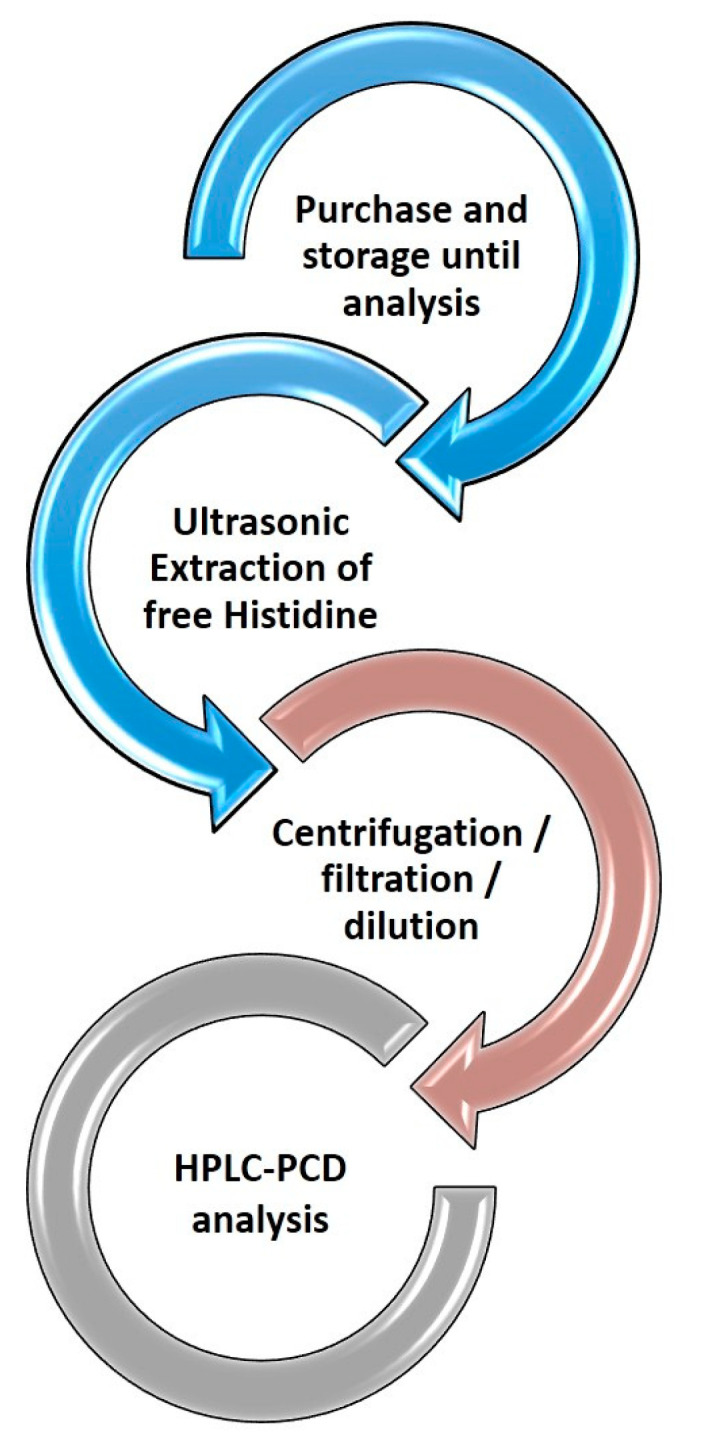
Representative workflow of the HPLC-PCD method for the determination of free histidine in hair care products.

Histidine is a polar compound, and its amino groups can be protonated in acidic media. For this reason, 5 mmol L^−1^ of HNO_3_ (mobile phase) was used for the extraction of the analyte in all cases, in this way matching the composition of the mobile phase and avoiding potential peak distortion phenomena. The extraction was promoted ultrasonically, and the extraction recovery was studied at time intervals of 15 and 30 min. The time of 30 min did not improve the extraction recovery, and it was concluded that 15 min was adequate to recover free histidine from the matrix. Following extraction, the samples were treated according to the Sample Preparation section (centrifugation, filtration and dilution). No foaming of the samples was observed at any stage. A graphical depiction of the sample treatment workflow is presented in Figure 2.

### 2.3. Analytical Figures of Merit

Linearity was obeyed in the range of 1.5–30 mg kg^−1^ histidine (r^2^ = 0.998) with LOD/LOQ = 0.5 and 1.5 mg kg^−1^, respectively. The mean within-day precision (expressed as % RSD) was 2.4% (*n* = 8 at 5 and 15 mg kg^−1^ histidine), while the between-day precision was 5.6% (as the RSD of the slopes, *n* = 6 non-consecutive days).

### 2.4. Study of the Matrix Effect

Due to the complexity of the samples, it was important to investigate the potential postextraction matrix effects [21,22]. The postextraction matrix effect was studied individually for the shampoo and conditioner products, using samples that did not contain endogenous histidine (according to the manufacturers’ labels). In brief, pooled shampoo and conditioner samples (*n* = 6 for each pooled matrix) were prepared individually and were treated as described in the experimental section. Each pooled matrix was spiked after extraction with histidine in the range of 5–20 mg kg^−1^. The matrix effect was calculated as the relative error of the slope of the individual matrix-matched calibration curves compared to the slope of the aqueous one. As shown in Table 2, due to the combination of cation-exchange chromatography and selective PCD, the percent postextraction matrix effects were negligible, being −1.1% and +3% for histidine in the shampoo and conditioner samples, respectively.

### 2.5. Analysis of Real Samples

**Figure 3:**
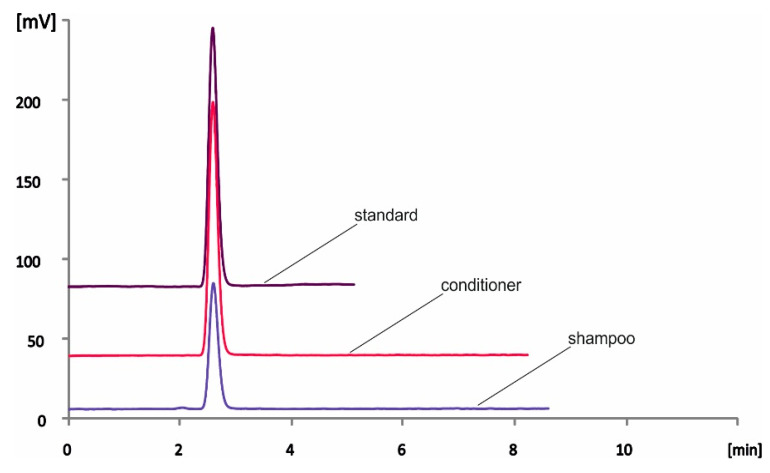
Representative overlaid chromatograms of standard solution (30 mg kg^−1^ histidine) and shampoo and conditioner samples using the proposed HPLC-PCD method.

The hair care samples were commercially available shampoo and conditioner products and were selected based on their labels’ claims. In the products that did not refer to histidine in their ingredients, the analyte was not detected. On the other hand, free histidine was quantified in all claimed samples, at concentration levels ranging between ca. 70 and 535 mg kg^−1^, as shown in Table 3. The dilution factor of each sample is shown in Table 3. Representative chromatograms from a standard solution and both shampoo and conditioner products are depicted in Figure 3. The accuracy of the method was evaluated by spiking experiments in the samples at two final concentration levels of 5 and 10 mg kg^−1^. As can be seen in Table 4, the recoveries were satisfactory and ranged between 83.5 and 114.8%.

## 3. Materials and Methods

### 3.1. Instrumentation

The HPLC instrumentation consisted of an AS3000 autosampler (Thermo Scientific, Walltham, MA, USA); a LC-9A binary pump (Shimadzu, Kyoto, Japan); and an Elite^TM^ vacuum degasser (Alltech, Athens, Greece). Chromatographic separations were performed using a MetroSep C4 column (150 × 4.0 mm i.d., 5 µm) (Metrohm, Herisau, Switzerland) thermostated at the desired temperature (Jones Chromatography oven). The PCD instrumentation consisted of a Minipuls^TM^ 3 peristaltic pump (Gilson, Middleton, WI, USA); the PCD reaction coil (200 cm, tightly knitted around a stainless-steel rod); and connections (made of PTFE tubing, i.d. = 0.5 mm). The reaction coil was thermostated at the required temperature (HiChrom Limited, Reading, UK). Detection was carried out using a RF-551 spectrofluorimetric detector operated at high sensitivity (λ_ex_/λ_em_ = 360/440 nm) (Shimadzu, Kyoto, Japan), and data acquisition was carried out via the Clarity^®^ software (version 4.0.3, DataApex, Prague, Czech Republic).

### 3.2. Reagents and Solutions

All reagents used in this study were commercially available and of analytical grade. The following reagents were used: histidine (Sigma-Aldrich), o-phthalaldehyde (OPA, Fluka), HNO_3_ (Fluka), KH_2_PO_4_ (Merk) and NaOH (Merck). Doubly deionized water was produced by a Milli-Q system (Millipore, Thessaloniki, Greece). The mobile phase consisted of 5 mmol L^−1^ HNO_3_ and was prepared daily, including ultrasonic degassing. The standard stock solution of histidine was prepared daily at the concentration level of 1000 μmol L^−1^ by dissolving accurately weighed amounts in the mobile phase. Working standard solutions were prepared by serial dilutions of the stock solution in the same solvent. The derivatizing reagent (OPA) was prepared at a concentration of 10 mmol L^−1^. Phosphate buffer was also prepared daily at 50 mmol L^−1^ and was adjusted to the pH value of 9 by addition of 2.0 mol L^−1^ NaOH solution.

### 3.3. HPLC-PCD Procedure

Standards or samples of 20 μL were separated through a cation-exchange column by isocratic elution (5 mmol L^−1^ HNO_3_) at a flow rate of 1.0 mL min^−1^ and a column temperature of 60 °C. The eluted compounds were mixed online with the PCD reagents at a flow rate of 0.25 mL min^−1^ for each stream. The derivatization reaction was allowed to proceed through a thermostated reaction coil (200 cm/60 °C), and the products were detected using the fluorescence detector at λ_ex_/λ_em_ = 360/440 nm.

### 3.4. Preparation of Samples

Two types of commercially available hair care products, namely, shampoos and conditioners, were purchased from local markets and were stored as for everyday use. A representative amount of 1.0 g of each product was weighed in a 15 mL plastic centrifuge tube and dispersed in 10 mL of HNO_3_ 5 mmol L^−1^. The extraction of histidine was promoted ultrasonically for 15 min. The mixture was centrifuged at 2000 rpm for 5 min, and the supernatant was filtered through a syringe filter. Each sample was analyzed at a preliminary step in order to determine the necessary dilution factor. Depending on the concentration of free histidine in the filtered solutions, the samples were appropriately diluted in the mobile phase prior to HPLC-PCD analysis. The validation of the method was based on histidine-free shampoo and conditioner matrices following the above pretreatment. Each sample was processed in triplicate.

## 4. Conclusions

In this study, the first analytical method was validated for the determination of free histidine in hair care products. Cation-exchange chromatography coupled to online postcolumn derivatization offers the determination of histidine in shampoo and conditioner samples, without matrix effects and with minimum sample preparation, at levels ranging between 70 and 535 mg kg^−1^.

## Figures and Tables

**Table 1:** Overview of recent analytical methods applied to hair care products.

Analyte(s)	Analytical Technique	Sample Preparation/Cleanup	Reference
Methyl and propyl parabens	UV spectrophotometry	Pipette tip micro-solid-phase extraction using chromium-based metal organic framework	[9]
N-nitrosodiethanolamine	Ultra-High-Pressure LC	Online solid-phase extraction using Oasis HLB	[10]
Zinc Pyrithione	Complexometric titration	Heating with HCl and H_2_O_2_	[11]
Dexpanthenol	Ultra-High-Pressure LC-MS	Dissolution–filtration–dilution	[12]
Zinc Pyrithione and Pyrithione	HPLC-MS/MS	Solvent extraction with chloroform/MeOH	[13]
N-nitrosodiethanolamine	HPLC-UV	Modified QuEChERS	[14]
Sulfur	UV-vis spectrophotometry	Dry ashing/wet digestion	[15]
Heavy metals	Atomic absorption spectrometry	Dry ashing/wet digestion	[16]
Ketoconazole	Ultra-High-Pressure LC-MS/MS	Solid-phase extraction using Oasis HLB	[17]
Fenoxycarb and Permethrin	HPLC-UV	Dissolution–filtration	[18]
Histidine	HPLC-PCD	Dissolution–filtration–dilution	This study

**Table 2:** Evaluation of the matrix effect (ME %) in shampoo and conditioner samples.

	Slope	ME %
Aqueous Curve	279.15 (±0.85)	
Shampoo Matrix	276.02 (±4.35)	−1.1%
Conditioner Matrix	287.42 (±3.65)	+3.0%

**Table 3:** Histidine content in shampoo and conditioner products (*n* = 3).

**Shampoo Samples**	**Histidine (mg** **k** **g^−1^) (±S.D.)**
Sample 1 ^a^	70 (±5)
Sample 2 ^a^	75 (±3)
Sample 3 ^b^	N.D.
**Conditioner Samples**	**Histidine (mg kg^−1^) (±S.D.)**
Sample 1 ^c^	270 (±10)
Sample 2 ^c^	145 (±5)
Sample 3 ^c^	300 (±12)
Sample 4 ^c^	535 (±13)
Sample 5 ^b^	N.D.

^a^ Samples were diluted 10-fold prior to analysis. ^b^ Samples were diluted 2-fold prior to analysis. ^c^ Samples were diluted 50-fold prior to analysis.

**Table 4:** Accuracy of the proposed method for the analysis of histidine in diluted real samples (*n =* 3).

**Shampoo Samples**	**Spiked (mg kg^−1^)**	**% Recovery**
Sample 1	5.0	103.5
	10.0	99.7
Sample 2	5.0	83.5
	10.0	91.4
Sample 3	5.0	97.3
	10.0	98.7
**Conditioner Samples**	**Spiked (mg kg^−1^)**	**% Recovery**
Sample 1	5.0	95.3
	10.0	98.0
Sample 2	5.0	110.3
	10.0	114.8
Sample 3	5.0	102.1
	10.0	97.0
Sample 4	5.0	95.3
	10.0	101.0
Sample 5	5.0	101.5
	10.0	102.5

## Data Availability

Data available upon request.

## Sample Availability

Samples are available from the authors.

## References

[1] Nogueira A.C.S., Dicelio L.E., Joekes I. (2006). About Photo-Damage of Human Hair. Photochem. Photobiol. Sci..

[2] Santos Nogueira A.C., Joekes I. (2004). Hair Color Changes and Protein Damage Caused by Ultraviolet Radiation. J. Photochem. Photobiol. B Biol..

[3] Marsh J.M., Iveson R., Flagler M.J., Davis M.G., Newland A.B., Greis K.D., Sun Y., Chaudhary T., Aistrup E.R. (2014). Role of Copper in Photochemical Damage to Hair. Int. J. Cosmet. Sci..

[4] Worasith N., Goodman B.A. (2013). Determination of the Coordination Environment of Cu(II) in Human Hair and Its Possible Relevance to Health and Hair Care Treatments. Int. J. Cosmet. Sci..

[5] Kizawa K., Inoue T., Yamaguchi M., Kleinert P., Troxler H., Heizmann C.W., Iwamoto Y. (2005). Dissimilar Effect of Perming and Bleaching Treatments on Cuticles: Advanced Hair Damage Model Based on Elution and Oxidation of S100A3 Protein. Int. J. Cosmet. Sci..

[6] Marsh J.M., Davis M.G., Flagler M.J., Sun Y., Chaudhary T., Mamak M., McComb D.W., Williams R.E.A., Greis K.D., Rubio L. (2015). Advanced Hair Damage Model from Ultra-Violet Radiation in the Presence of Copper. Int. J. Cosmet. Sci..

[7] Pan Y., Zhang L.Y., Liu Y.Z. (2013). XAFS Study of Coordination Structure of Cu(L-His)2 in Solution. Chin. J. Chem. Phys..

[8] Drommi M., Rulmont C., Esmieu C., Hureau C. (2021). Hybrid Bis-Histidine Phenanthroline-Based Ligands to Lessen A&beta;-Bound Cu ROS Production: An Illustration of Cu(I) Significance. Molecules.

[9] Abedi G., Talebpour Z. (2017). Modified QuEChERS as a Novel Sample Preparation Method for Analysis of: N -Nitrosodiethanolamine in Shampoo by High Performance Liquid Chromatography. Anal. Methods.

[10] Bielemann N.J., Novo D.L.R., Pereira R.M., Mello J.E., Costa V.C., Mesko M.F. (2017). Determinação De Enxofre Em Shampoo Por Espectrofotometria Uv-Vis: Avaliação De Métodos De Preparo De Amostras. Quim. Nova.

[11] Egurrola G.E., Mazabel A.P., García J. (2021). Development and Validation of a Complexometric and Potentiometric Titration Method for the Quantitative Determination of Zinc Pyrithione in Shampoo. J. Anal. Methods Chem..

[12] Islam D.J. (2015). Determination of Heavy Metals and Trace Elements in Worldwide Branded Shampoo Available in Local Market of Bangladesh by Atomic Absorption Spectrometry. Asian J. Chem..

[13] Kaykhaii M., Hashemi S.H., Andarz F., Piri A., Sargazi G., Boczkaj G. (2021). Chromium-Based Metal Organic Framework for Pipette Tip Micro-Solid Phase Extraction: An Effective Approach for Determination of Methyl and Propyl Parabens in Wastewater and Shampoo Samples. BMC Chem..

[14] Kim S.H., Shrestha A., Hoang N.H., Huong N.L., Park J.W. (2014). Ultra-Performance Liquid Chromatography with Electrospray Ionization Tandem Mass Spectrometry for the Determination of Ketoconazole in Anti-Dandruff Shampoo. Anal. Lett..

[15] Kim T.H., Jung G.H., Lee E.H., Park H.R., Lee J.K., Kim H.G. (2018). Development and Validation of Liquid Chromatography–Tandem Mass Spectrometry Method for Simultaneous Determination of Zinc Pyrithione and Pyrithione in Shampoos. Acta Chromatogr..

[16] Šatínský D., Kameníčková D., Chocholouš P., Solich P. (2013). Fast HPLC Method for Determination of Fenoxycarb and Permethrin in Antiparasitic Veterinary Shampoo Using Fused-Core Column. Chromatographia.

[17] Tada A., Rodrigues-Silva C., Rath S. (2021). On-Line Solid Phase Extraction-Ultra-High Performance Liquid Chromatography Coupled to Tandem Mass Spectrometry for the Determination of N-Nitrosodiethanolamine in Baby Shampoo. J. Pharm. Biomed. Anal..

[18] Weiss C.L., Fairchild M.R., Stanton B., Nshime B.S., Parkanzky P.D. (2019). Innovative Method for the Analysis of Dexpanthenol in Hair Care Products. J. AOAC Int..

[19] Alevridis A., Tsiasioti A., Zacharis C.K., Tzanavaras P.D. (2020). Fluorimetric Method for the Determination of Histidine in Random Human Urine Based on Zone Fluidics. Molecules.

[20] Stampina E., Tsiasioti A., Klimatsaki K., Zacharis C.K., Tzanavaras P.D. (2021). Determination of Histidine in Human Serum and Urine by Cation Exchange Chromatography Coupled to Selective On-Line Post Column Derivatization. J. Chromatogr. B.

[21] Kumagai M., Kato S., Arakawa N., Otsuka M., Hamano T., Kashiwagi N., Yabuki A., Yamato O. (2021). Quantification of Histidine-Containing Dipeptides in Dolphin Serum Using a Reversed-Phase Ion-Pair High-Performance Liquid Chromatography Method. Separations.

[22] Gkantiri A.M., Tsiasioti A., Zacharis C.K., Tzanavaras P.D. (2022). HPLC Method with Post-Column Derivatization for the Analysis of Endogenous Histidine in Human Saliva Validated Using the Total-Error Concept. Amino Acids.

